# Prolactin-Induced Protein (PIP) Regulates Proliferation of Luminal A Type Breast Cancer Cells in an Estrogen-Independent Manner

**DOI:** 10.1371/journal.pone.0062361

**Published:** 2013-06-03

**Authors:** Sanjeev K. Baniwal, Nyam-Osor Chimge, V. Craig Jordan, Debu Tripathy, Baruch Frenkel

**Affiliations:** 1 Department of Orthopedic Surgery, Keck School of Medicine of the University of Southern California, Los Angeles, California, United States of America; 2 Institute for Genetic Medicine, Keck School of Medicine of the University of Southern California, Los Angeles, California, United States of America; 3 Department of Biochemistry and Molecular Biology, Keck School of Medicine of the University of Southern California, Los Angeles, California, United States of America; 4 Department of Oncology, Breast Cancer Program, Georgetown-Lombardi Comprehensive Cancer Center, Georgetown University Medical Center, Washington, D.C., United States of America; 5 Department of Medicine, Keck School of Medicine of the University of Southern California, Los Angeles, California, United States of America; H. Lee Moffitt Cancer Center & Research Institute, United States of America

## Abstract

Prolactin-induced Protein (PIP), an aspartyl protease unessential for normal mammalian cell function, is required for the proliferation and invasion of some breast cancer (BCa) cell types. Because PIP expression is particularly high in the Luminal A BCa subtype, we investigated the roles of PIP in the related T47D BCa cell line. Nucleic acid and antibody arrays were employed to screen effects of PIP silencing on global gene expression and activation of receptor tyrosine kinases (RTKs), respectively. Expression of PIP-stimulated genes, as defined in the T47D cell culture model, was well correlated with the expression of PIP itself across a cohort of 557 mRNA profiles of diverse BCa tumors, and bioinformatics analysis revealed cJUN and cMYC as major nodes in the PIP-dependent gene network. Among 71 RTKs tested, PIP silencing resulted in decreased phosphorylation of focal adhesion kinase (FAK), ephrin B3 (EphB3), FYN, and hemopoietic cell kinase (HCK). Ablation of PIP also abrogated serum-induced activation of the downstream serine/threonine kinases AKT, ERK1/2, and JNK1. Consistent with these results, PIP-depleted cells exhibited defects in adhesion to fibronectin, cytoskeletal stress fiber assembly and protein secretion. In addition, PIP silencing abrogated the mitogenic response of T47D BCa cells to estradiol (E2). The dependence of BCa cell proliferation was unrelated, however, to estrogen signaling because: 1) PIP silencing did not affect the transcriptional response of estrogen target genes to hormone treatment, and 2) PIP was required for the proliferation of tamoxifen-resistant BCa cells. Pharmacological inhibition of PIP may therefore serve the bases for both augmentation of existing therapies for hormone-dependent tumors and the development of novel therapeutic approaches for hormone-resistant BCa.

## Introduction

Prolactin-induced Protein (PIP), a.k.a. serum actin-binding protein (SABP) and gross cystic fluid protein (GCDFP)-15, is a ∼15 KDa glycoprotein expressed by a majority of breast cancer (BCa) tumors [1]. Its expression is particularly high in the luminal A and androgen receptor (AR)-positive HER2-enriched breast cancer subtypes [2], [3]. PIP is also biosynthesized and secreted by a number of normal apocrine cell types that produce milk, seminal fluid, tear, and saliva [1]. In addition to prolactin, PIP is induced by androgens, growth hormone and glucocorticoids [4], [5]. In T47D BCa cells, 5α-dihydrotestosterone (DHT) at physiological concentrations was most potent inducer, increasing PIP expression by >12-fold [4], [6], [7]. Furthermore, immunohistochemical staining of BCa tumors suggested a strong correlation between the expression levels of PIP and the androgen receptor (AR), as well as between PIP and prostate-specific antigen (PSA), a classical AR-regulated gene [2]. Hormone stimulated expression of PIP requires Runx2, a pro-metastatic transcription factor. Co-recruitment of AR and Runx2 to an enhancer located ∼11 Kb upstream of the PIP transcription start site [8] and the physical interaction between these two transcription factors [9], likely mediate synergistic stimulation of PIP expression. In turn, PIP formed a feed-forward loop by enhancing AR signaling [8]. Recently, an additional positive feedback loop was identified where PIP was required for the recruitment of CREB1 to the proximity of the PIP transcription start site [3].

Despite widespread expression, the function of PIP in both normal and cancer cells remains obscure. PIP deficient mice are essentially normal indicating that its function under physiological conditions is either non-essential or complimented by other protein/s. In contrast to normal cells, treatment of various human BCa cell lines with purified PIP enhanced their proliferation [10] and PIP silencing in both ERa-positive and ERa-negative BCa cell lines inhibited cell proliferation as well as invasion through an artificial extracellular matrix [3], [8]. These studies indicate that PIP acquires an essential function during cellular transformation. Potentially related to this function is its aspartyl protease activity, which was demonstrated using purified PIP and fibronectin as the substrate. The resultant fibronectin fragments bound integrin beta-1 receptors and activated signaling pathways related to BCa cell proliferation and invasion [3], [11].

In pursuit of PIP-dependent signaling pathways that regulate BCa cell proliferation, we employed PIP knock down and high throughput mRNA profiling as well as antibody arrays to identify gene networks and receptor tyrosine kinases (RTKs) that execute PIP's function. The results suggest that PIP is required for the activation of specific RTKs, including FAK. Accordingly, we demonstrate a role of PIP in fibronectin adhesion and in cytoskeleton dynamics. Finally, we demonstrate requirement for PIP for the proliferation of tamoxifen-resistant BCa cells, suggesting that PIP may be targeted for the development of novel therapeutic approaches to treat BCa patients who do not respond to hormonal therapy.

## Methods

### Cell culture

ER-positive T47D and ZR-75 and ER-negative MDA-MB-453 cells were from American Type Culture Collection (ATCC). T47D cells were maintained in RPMI-1640, and MDA-MB-453 and ZR-75 cell lines in DMEM medium, both supplemented with 10% fetal bovine serum from Clontech, CA. Before hormone treatment cells were washed three times with PBS and maintained for 48 hours in phenol-red free growth medium supplemented with 10% charcoal-stripped serum (CSS). Tamoxifen resistant T47D cells (T47D^tamR^) were derived earlier by long-term growth of cells in 1 µM 4- hydroxytamoxifen (4-OHT) [12], [13]. The growth medium for T47D^tamR^ cells was further supplemented with 1 µM 4-OHT, 10 mM non-essential amino acids, 200 mM L-glutamin, and 10 microgram/ml insulin.

### Antibodies and Reagents

The anti-PIP antibody (ab 62363) was purchased from abcam Inc., Cambridge, MA. The antibodies for detecting total and phosphorylated form of AKT (9272, 9275), ERK1/2 (4695, 9101), and HGFR (3148, 13D11) were purchased from Cell Signaling Technology, Inc., Danvers, MA. Total JNK1/2 levels were detected by JNK antibody (sc-572) from Santa Cruz (Biotechnology Inc., Santa Cruz, CA. Levels of [pThr^183^/Tyr^185^]-JNK1/2 were assessed using phosphor-SAPK/JNK (4668) antibody from Cell Signaling Technology, Inc. The Anti-FAK antibody, clone 4.47 and PhosphoDetect™ Anti-FAK (pTyr^397^) were purchased from Millipore, Billerica, MA. Antibodies detecting total Myc and [pThr^58^/pSer^62^]-Myc were from Cell Signaling Technology, Inc. The mouse monoclonal anti- β -tubulin antibody, developed by Dr. Charles Walsh, was obtained from the Developmental Studies Hybridoma Bank under the auspices of the NICHD and The University of Iowa, Department of Biological Sciences, Iowa City, USA. The hormones 17β-estradiol (E2) and 4- OHT, and insulin were purchased from from Sigma, St Louis, MO. E2 and tamoxifen were used at a concentration of 30 nM and 1 microM, respectively. Equal volume of ethanol was used as vehicle control. The growth media RPMI-1640 and DMEM, as well as the supplemental non-essential amino acids and L-glutamine were purchased from Gibco, Grand Island, NY. Hygromycin B was purchased from Invitrogen, Carlsbad, CA, USA, and added to the growth medium at 50 µg/ml. Doxycycline from Calbiochem, La Jolla, CA was used at 250 ng/ml unless otherwise indicated. An equal volume of distilled water was used as vehicle control. Puromycin, 3-(4,5-Dimethylthiazol-2-yl)-2,5-diphenyltetrazolium bromide (MTT) was obtained from Sigma, St Louis, MO. Phalloidin powder for the visualization of actin stress fiber was purchased from Enzo Life Sciences, Inc., Farmingdale, NY. A stock solution was prepared by dissolving 0.1 mg of phalloidin powder in 1 ml of dimethyl sulfoxide (DMSO), and a working solution was prepared by a further 1∶2000 dilution in PBS.

### Plasmids

The dox-inducible lentiviral plasmids were based on the pSLIK (single lentivector for inducible knockdown) vector [23]. DNA sequences encoding shRNAs for PIP were designed using the RNAiCodex program (http://katahdin.cshl.org/html/scripts/resources.pl). Oligonucleotides used for cloning are listed in Table S1. The shRNA-coding oligonucleotides were initially cloned into the lentiviral entry vector pEN_TmiRc3 (ATCC® catalog: MBA-248), and the resulting plasmid was recombined using Gateway® LR Clonase® II enzyme mix (Invitrogen) with the pSLIK destination vector carrying a hygromycin resistance gene (ATCC® catalog: MBA-237). Constitutively expressing shRNA lentiviral plasmids targeting either a non-specific sequence or distinct PIP-specific sequences were purchased from Sigma (Table S1). The constitutively expressed shPIP/121 and shPIP/214 hairpin RNAs target the nucleotide sequences 121–140 and 214–230 within the PIP open reading frame (ORF), respectively. Dox-inducible shRNA targeted either nucleotides 263–283 of the PIP ORF or nucleotides 32–52 of the 3′UTR, the latter used in the co-culture assays only (Table S1).

### RNA extraction, RT-qPCR, and analysis of global gene expression

RNA was extracted from cells using Aurum Total RNA kit from Bio-Rad Laboratories, Inc., Hercules, CA following the manufacturer's recommendations. For cDNA synthesis, 1 microgram of RNA was processed using qScript™ cDNA SuperMix as per the manufacturer's instructions (Quanta BioSciences, Inc., Gaithersburg, MD). The cDNA was diluted 10-fold with distilled water and subjected to real-time PCR amplification using Maxima® SYBR Green/Fluorescein qPCR Master Mix (2×) from Fermentas Inc., Glen Burnie, MD and CFX96™ RT-PCR system from Bio-Rad, Hercules, CA. The sequences of primers used for real-time PCR amplification are listed in Table S1. Gene expression profiling was performed using the BeadChip HumanHT-12 v4 Expression kit from Illumina®, which contains 47,231 gene-probes (Illumina® Inc., San Diego, CA). The raw signal intensities were imported and analyzed using the GenomeStudio® data software. After background subtraction and normalization, the signal intensity values were exported to the Partek® genomics expression analysis suite using “Partek's Report Plug-in” option in the GenomeStudio® software. Differentially expressed genes in the dox- *versus* vehicle-treated samples were identified using the “gene expression” workflow in the Partek® software. The differentially expressed probes were further investigated using the Ingenuity Pathways Analysis package (IPA™; http://www.ingenuity.com) to identify the association of differentially expressed genes with “disease and disorder” and “molecular and cellular functions” categories. Right-tailed Fisher's exact test as implemented in the IPA software was used to calculate a p-value for the probability of each network to be enriched for PIP-regulated genes due to chance alone. The microarray data has been deposited in the GEO database with the accession code GSE41894.

### Lentivirus production

For packaging, the lentiviral expression plasmids were co-transfected by the calcium chloride method into HEK293T cells along with helper plasmids pMD.G1 and pCMVΔR8.91 [24], [25]. Culture media containing viral particles were harvested after 48–72 hours and used for transduction of the indicated cells in the presence of 8 µg/ml Polybrene (Millipore Corp., MA, USA). The transduced cells were selected with either 50 µg/ml of Hygromycin or 3 microgram/mL of Puromycin.

### Cell proliferation assay

Cell proliferation was assessed using either MTT- or luciferase-based assays. For MTT assays, cells were incubated at 37°C with 0.5 mg/mL of MTT dissolved in PBS for 2 hours. Cells were then lysed using DMSO and the development of color was quantified at 595 nM using Victor_3_V™ from PerkinElmer, Shelton, CT, USA. For luciferase assays cells were first transduced with lentiviral particles containing a constitutive green fluorescent protein (GFP)/luciferase cassette to facilitate their isolation using fluorescence activated cell sorting and to later assess their proliferation based on luciferase activity. Approximately 5,000 fluorescently-sorted cells were plated in a 24 well tissue culture plates in the presence of dox or an equal volume of water as vehicle control. Samples were harvested every 48 hours by lysing the cells in 200 microlitre of passive lysis buffer purchased from Promega, Madison, WI, and were stored at −80°C until quantification of luciferase activity using Victor_3_V™.

### Receptor Tyrosine Kinase Screen

We used the Human RTK Phosphorylation antibody array 1 kit (RayBiotech, Inc., Norcross, GA), which facilitates simultaneous detection of the phosphorylation status of 71 major receptor tyrosine kinases. T47D cells were transduced with lentiviruses encoding shRNA specific for either PIP (sh121) or a non-genomic DNA sequence (Table S1). Preparation of cell lysates as well as hybridization to the membrane containing the dedicated spots for 71 receptor tyrosine kinases and controls was conducted as per the manufacturer's recommendations. Briefly, 250 μg of cell lysate proteins was diluted in 1.2 ml blocking buffer and incubated for 2 hours at 4°C with gentle shaking. The membranes were washed and further incubated with biotin-conjugated antibodies that bind phosphotyrosine residues. To enable detection of phosphotyrosine-bound antibodies, the membranes were washed and incubated with biotinylated antiphosphotyrosine antibodies. Washed membranes were subjected to chemiluminescence-based imaging using the Western Lightning™ Plus-ECL kit from PerkinElmer Inc, Waltham, MA and then exposed to Clear Blue X-ray Film from Bioland Scientific LLC, Paramount, CA.

### Cell adhesion assay

The T47D/shNS/LUC, T47D/shPIP/121/LUC and T47D/shPIP/214/LUC cells encoding either non-specific (shNS) or PIP-specific (shPIP shPIP/121, shPIP/214) shRNAs, respectively, and a constitutively active luciferase gene were cultured for 2 days in the presence or absence of dox (250 ng/mL). Quadruplicates of about 10,000 cells were plated in a 24 well plate pre-coated for 1 hour at 37°C with 10 microgram/ml of either fibronectin or bovine serum albumin as background control (both from Sigma). After 1 hour of incubation, unbound cells were washed three times with PBS and the relative numbers of bound cells were assessed based on luciferase assays. The arginine-glycine-aspartate (GRGDSP) and arginine-glycine-glutamic acid (GRGESP) peptides were obtained from Anaspec, Inc., San Jose, CA.

### Visualization of stress fibers by phalloidin staining

To visualize stress fibers, cells were grown on fibronectin-coated slides for 48 hours in the presence or absence of dox. Cells were washed three times with phosphate buffered saline and incubated for one hour in cell culture incubator in growth medium without serum. Cells were fixed with chilled 0.5% paraformaldehyde solution, permeabilized with chilled 0.1% Triton X-100 in PBS, and incubated in phalloidin solution for 1 hour at room temperature. Stress fibers were visualized using a Zeiss LSM 510 inverted confocal microscope and the Zeiss LSM Image Browser software (Carl Zeiss Inc., Jena, Germany) for image stacking.

### Cell extract preparation and western blot analysis

Whole cell extracts were prepared by lysing 1×10^5^–2×10^5^ cells in 200 microlitre of lysis buffer [100 mM Tris (pH 7.4), 500 mM NaCl, 1 mM CaCl_2_, 0.5 mM MgCl_2_, 0.1% Nonidet® P-40] supplemented with Complete™ protease inhibitor mix (Roche Diagnostics, Indianapolis, IN). Thirty microgram protein was mixed with an equal volume of 2× Laemmli buffer followed by SDS-PAGE. Electrophoresed proteins were transferred to Amersham Hybond™-P PVDF membranes (GE Healthcare, Piscataway, NJ), and detected using specific antibodies and the Western Lightning™ Plus-ECL kit.

## Results

### PIP is preferentially expressed in breast cancer cells of the Luminal A subtype and is required for their proliferation independently of its secretion

**Figure 1:**
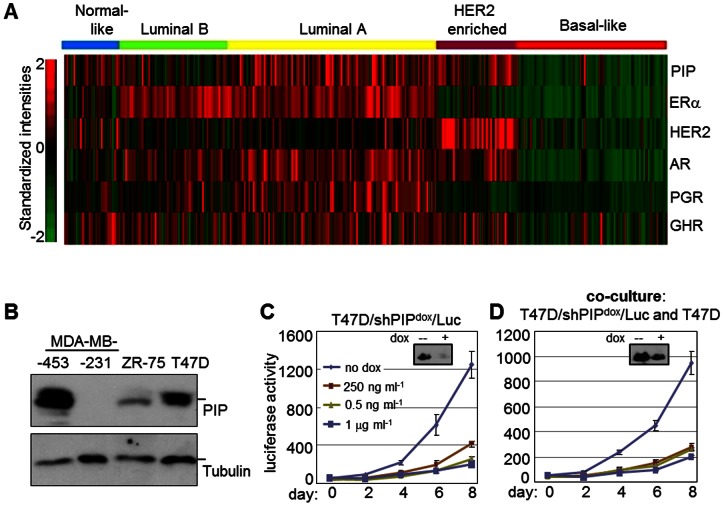
Relative expression of PIP in intrinsic breast cancer subtypes and requirement of intracellular PIP for cell proliferation. **A**, Heat map demonstrating the mRNA expression levels of PIP and various hormone receptors known to regulate its expression in a heterogeneous cohort of 557 BCa patients. The patient cohort was compiled from the publicly available GEO datasets GSE2034, GSE2603 and GSE12276 and was described previously [8]. The intrinsic subtype of each tumor was determined according to the PAM-50 algorithm [36]. Expression values for the indicated genes were derived from the signal intensities associated with the following affymetrix probesets: 206509_at (PIP), 205225_at (ESR1) 216836_s_at (HER2), 211110_s_at (AR), 208305_at (PGR) and 205498_at (GHR). **B**, Immunoblot analysis of PIP in the ER-alpha positive BCa cell lines MDA-MB-453, ZR-75 and T47D and in the ER–negative MDA-MB-231 BCa cell line. Tubulin was analyzed as control. **C–D**, Luciferase assays were performed at the indicated days after plating to assess the proliferation of T47D/PIP^dox^/LUC cells cultured in serum-supplemented medium either alone (*C*) or together with naïve T47D cells (*D*). The co-cultures were started with roughly equal number (∼5,000) of T47D/shPIP^dox^/LUC and naïve T47D cells. Doxycycline (dox) was added at the time of plating at the indicated concentrations. Insets in *C* and *D* show PIP levels by immunoblotting after 4 days of treatment with either vehicle or 250 ng/mL dox.

PIP is expressed by a majority of BCa tumors and serves as a marker for disease progression [1]. Breast tumors are heterogeneous in nature with at least five well-recognized intrinsic molecular subtypes that differ in clinical progression and drug responsiveness: basal-like, HER2-enriched, luminal A, luminal B, and normal-like [14], [15], [16]. In order to select a relevant BCa cell line for this study, we assessed its expression with respect to tumor subtypes using mRNA profiles of 557 breast tumors compiled from the three microarray datasets GSE2034, GSE7390, and GSE11121 [8], [17], [18], [19]. PIP mRNA was most highly expressed in the luminal A subtype, followed by HER2-enriched and normal-like tumors, with the least expression observed in basal-like tumors (Figure 1A). Consistent with these results, western blot analysis (Figure 1B) readily detected PIP expression in the T47D and MDA-MB-453 cell lines that represent the luminal A and HER2 subtypes, respectively [20]. In contrast, PIP expression was very low or absent in the ZR-75 and MDA-MB-231 cell lines (Figure 1B) that represent the luminal B and basal subtypes, respectively [21]). In subsequent experiments, we therefore pursued the role of PIP in BCa cells by knocking it down in the luminal A-like T47D cell line.

We recently reported that PIP was required for the proliferation of breast cancer cells [8]. Although PIP is best known as a secreted protein, follow-up experiments showed that PIP-containing conditioned medium from naïve T47D cells did not rescue the growth arrest imposed by PIP knockdown (data not shown). This suggested that PIP was likely required for a critical intracellular cell cycle regulatory function(s). To directly address this notion, we measured the proliferation of T47D/shPIP^dox^/LUC cells cultured either alone or together with naïve T47D cells (Figure 1C, D). The T47D/shPIP^dox^/LUC cells are transduced with dox-inducible shRNA specifically targeting the PIP ORF [8]; Table S1). In addition, the cells are marked with a constitutively active luciferase gene, so that their growth can be monitored by following accumulation of luciferase activity per well over time. As shown in Figure 1C, dox treatment of the T47D/shPIP^dox^/LUC cell cultures resulted in PIP silencing and subsequent dose-dependent inhibition of cell proliferation. In contrast to this simple culture system, when the T47D/shPIP^dox^/LUC cells are co-cultured with naïve T47D cells (Figure 1D), the latter continue to secrete PIP even in the presence of dox, and could act in a paracrine manner to rescue proliferation of the dox-treated T47D/shPIP^dox^/LUC cells. However, the dox-induced growth inhibition was equally apparent with and without co-cultured naive T47D cells, suggest that PIP was required for a novel intracellular function independent of its secretion.

### Analysis of global gene expression indicates a role for PIP in stimulating a highly connected JUN/MYC-centered transcriptome

**Figure 2:**
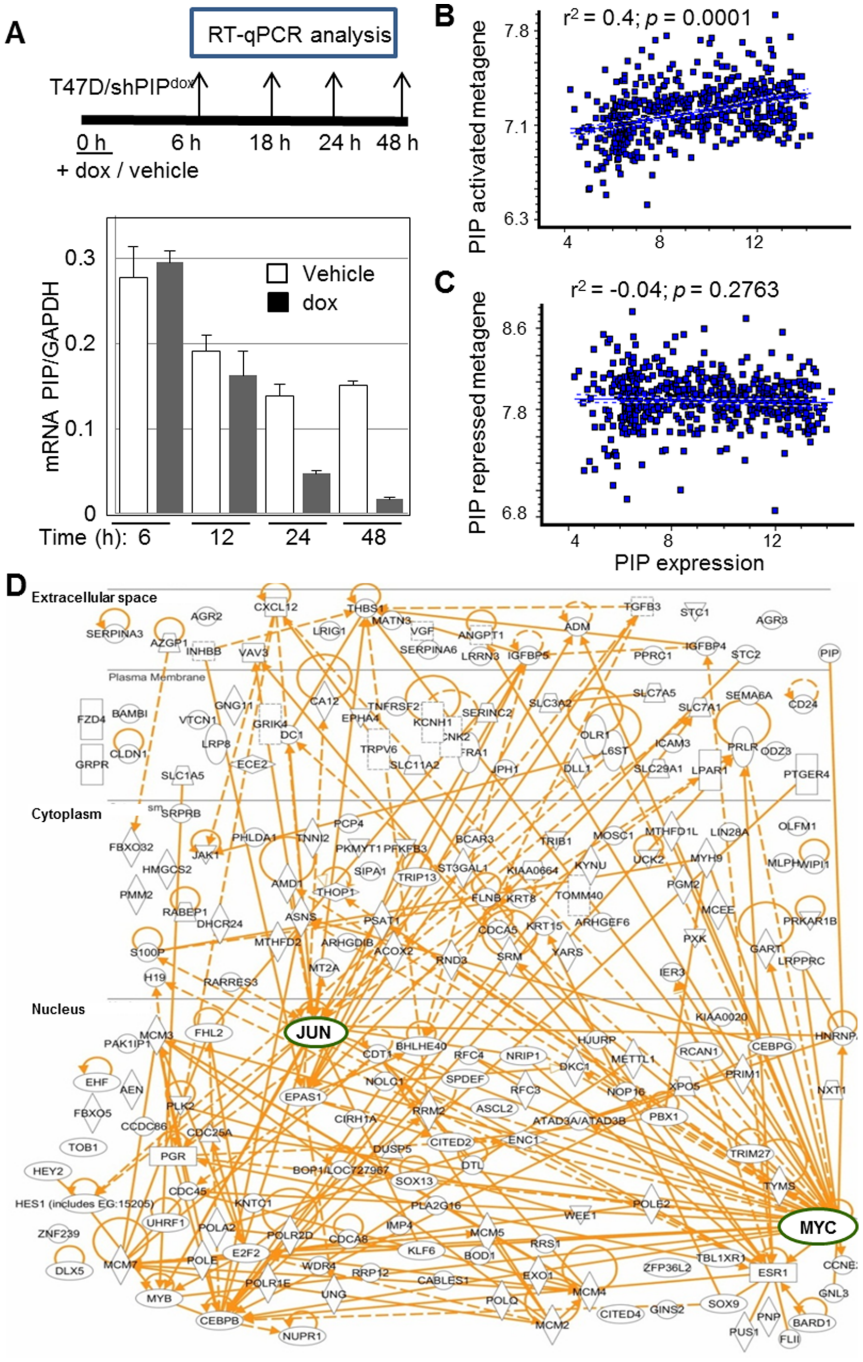
Identification of PIP-regulated genes in breast cancer. **A,** T47D/shPIP^dox^ cells were treated with dox (250 ng/ml) or vehicle, and RNA was extracted at four time points as schematically illustrated in the *upper panel*. RT-qPCR analysis of PIP with GAPDH as control (*bottom panel*) demonstrates effective PIP knockdown at the 24 and 48, but not at the 6 or 12-hour time points. **B–C,** Analysis of the clinical correlations in a cohort of 557 BCa tumors between PIP mRNA and either the PIP-activated (B) or the PIP-repressed metagenes (C), defined as the average normalized expression of genes that were either repressed (B) or stimulated (C) by ≥2-fold in the T47D/shPIP^dox^ cell culture model. **D**, 293 genes down-regulated by ≥1.5-fold one day after PIP knockdown were subjected to the pathway analysis tool from Ingenuity Systems (IPA™). Connections are shown among 200 genes for which matched entries were found. Each connection indicates at least one direct relationship found in the literature. Circles denote the highly connected cMYC and cJUN nodes.

In pursuit of PIP-regulated gene networks mediating its function(s) in BCa cells, we initially investigated changes in global gene expression in response to dox-mediated knockdown of PIP in T47D/shPIP^dox^ cells. A time course experiment demonstrated that dox treatment knocked down PIP mRNA by 70% and 80% at the 24 h and 48 h time points, respectively (Figure 2A). We note that PIP mRNA expression was also responsive to serum, but the serum response and effects of the PIP shRNA appear unrelated (Figure S4 in File S1). Because dox did not significantly inhibit PIP expression at the earlier 6 h and 12 h time points, we reasoned that mRNA profiling of cells after 24 h and 48 h of dox treatment would disclose primary responses to PIP knockdown. Cells were treated and their mRNAs analyzed in biological triplicates (a total of 12 samples) using Illumina's HumanHT-12 v4 BeadChips. Dox treatment differentially regulated the expression of 1,356 genes by ≥1.5-fold (690 repressed, 666 induced) at the 24 h or 48 h time points in a statistically significant manner (*p*<0.04; Table S2). RT-qPCR analysis of five randomly selected genes conformed to the microarray data (data not shown). Unsupervised hierarchical clustering of the most differentially regulated genes showing ≥2-fold effects at either 24 or 48 hours resulted in a clear separation between the dox-treated and control samples (Figure S1 in File S1). In general, the variation among the biological triplicates was small, and changes observed after 24 hours of treatment were maintained or intensified by 48 hours of treatment.

To examine the clinical relevance of our *in vitro* results, we interrogated publicly available RNA expression datasets for correlation between expression of *PIP* and that of genes that were down- or up-regulated by PIP silencing in the T47D cell culture model. We defined a PIP-activated metagene as the average normalized expression of genes that were most downregulated (≥2-fold; 41 genes, Figure S1 in File S1) after dox-mediated PIP knockdown in T47D/shPIP^dox^ cells. A PIP-repressed metagene was similarly defined as the average normalized expression of 41 genes most upregulated after dox-mediated PIP knockdown in T47D/shPIP^dox^ cells. A scatter plot of PIP expression *versus* that of the PIP-activated metagene across 557 BCa tumors revealed a highly significant positive correlation (r^2^ = 0.4; *p*<0.0001), suggesting that in a clinical setting, PIP most likely regulates genes comprising the PIP-activated metagene as defined in the T47D/shPIP^dox^ culture system (Figure 2B). In contrast, there was no significant correlation across the BCa tumors between expression of *PIP* and that of the PIP-repressed metagene (Figure 2C), suggesting that changes in the expression of these genes may represent secondary *in vitro* effects of PIP knockdown that are irrelevant *in vivo*. We therefore went on to investigate the potential significance of the PIP-activated metagene.

We employed the Ingenuity Pathway Analysis (IPA™) software package to gain insight into the most likely biological roles of the PIP-activated metagene. The “IPA Pathways and Path Designer” platform visualizes connections between differentially regulated genes based on published articles that document functional interactions between genes. Analysis of the genes down-regulated by PIP knockdown revealed an intricate network with cMYC and cJUN, master transcriptional regulators of cell proliferation, forming two central hubs (Figure 2D). The high connectivity of the PIP-activated metagene contrasts the low connectivity between genes comprising the PIP-repressed metagene (Figure S2 in File S1), and suggests that the PIP-activated genes represent a network(s) with a well-established function(s). To identify molecular events that occur immediately after PIP knockdown we used a set of 293 genes that were down-regulated by ≥1.5-fold with high statistical significance (p<0.04) after 24 hours of dox treatment (Table S2). IPA analysis of this gene-set showed that “cellular growth and proliferation”, “cell cycle” and “DNA replication, recombination, and repair” were the molecular and cellular functions most significantly associated with PIP (*p*<1.3E^−02^; Table 1). In particular, the most PIP-responsive genes encoding nuclear proteins (Tables 2, S3) are clearly enriched for such with well established roles in promoting cell proliferation, including cMYC, cJUN, E2F2, MYB and MCM5 [22], [23], [24], [25], [26].

**Table 1:** Deduced Molecular and Cellular Functions of PIP in T47D/shPIP^dox^ cells.

Function	p-value	# Molecules
Cellular Growth and Proliferation	1.3E^−10^ −1.3E^−02^	98
Cell Cycle	4.5E^−08^ −1.3E^−02^	51
DNA Replication, Recombination, and Repair	1.6E^−07^ −1.3E^−02^	39

The 293 genes repressed by ≥1.5 fold after one day of dox-induced PIP knockdown were analyzed using the IPA software package for enriched Molecular and Cellular Functions.

**Table 2:** PIP-regulated genes encoding nuclear proteins.

Gene ID	Entrez gene name	Fold repression
MCM5	Minichromosome maintenance complex component 5	2.3
RRM2	Ribonucleotide reductase M2	2.3
E2F2	E2F transcription factor 2	2.2
SPDEF	SAM pointed domain containing ets transcription factor	2.2
MYB	v-myb myeloblastosis viral oncogene homolog	2.2
CABLES1	Cdk5 and Abl enzyme substrate 1	2.1
UHRF1	Ubiquitin-like with PHD and ring finger domains 1	2.0
MYC	v-myc myelocytomatosis viral oncogene homolog	2.0
JUN	Jun proto-oncogene	2.0
SOX9	SRY (sex determining region Y)-box 9	1.9

PIP was silenced in T47D/shPIP^dox^ cells by dox treatment, and mRNA was globally profiled after 24 hours using BeadChip HumanHT-12 v4 Expression kit from Illumina®. Listed are the ten gens encoding nuclear proteins (as per Ingenuity Pathways Analysis package from IPA™), which were most repressed in the dox- versus vehicle-treated cells. The most repressed genes encoding extracellular, membrane, and cytoplasmic proteins are listed in Table S3.

### PIP is required for the activation of major receptor tyrosine kinases (RTKs) and their downstream kinase effectors AKT and ERK1/2

**Figure 3:**
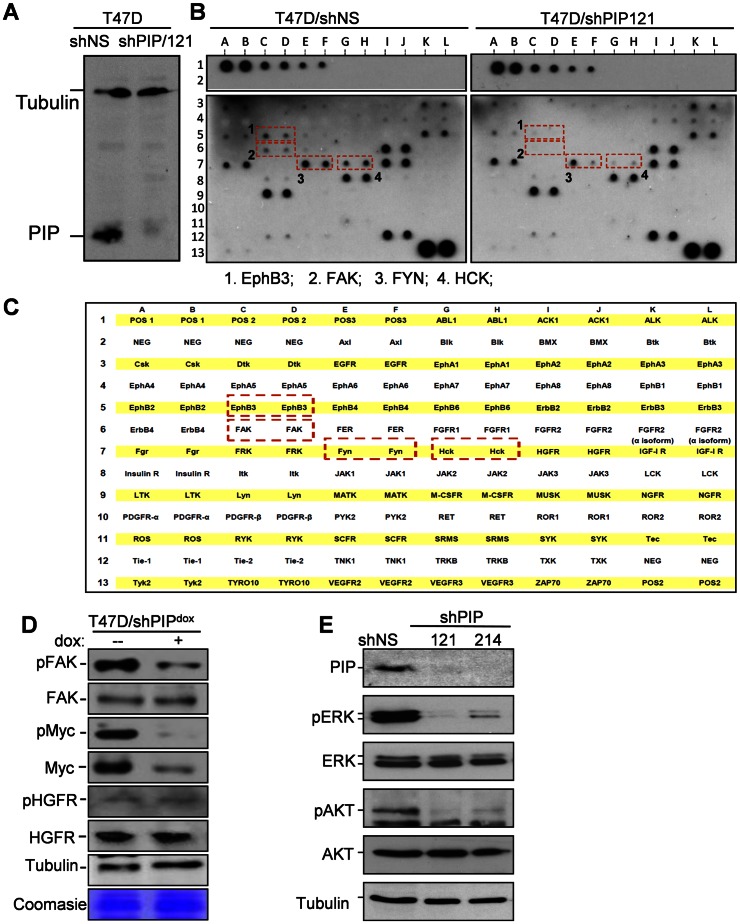
PIP silencing reduces phosphorylation of FAK, EphB3, FYN and HCK, and the downstream AKT and ERK1/2. **A,** Immunoblot analysis of PIP in T47D cells expressing shRNA against PIP (shPIP/121) or a non-genomic target (shNS). **B–C,** Receptor tyrosine kinase (RTK) phosphorylation screen (B), and a map of the 71 RTKs and controls spotted on the antibody array (C). Cell extracts from T47D/shPIP/121 and T47D/shNS cells were hybridized to the membranes and RTKs showing reduced phosphorylation in PIP-silenced cells are indicated by red boxes. **D,** Western blot analysis of T47D/shPIP^dox^ cells grown in serum-supplemented medium with dox or vehicle control. Antibodies were against [pTyr^397^]-FAK and pan FAK, [pThr^58^/pSer^62^]-Myc and pan Myc, as well as [Tyr^1003^]-HGFR and pan HGFR. Immunoblot of tubulin and a random coomasie blue-stained band are shown as loading controls. **E,** Western blot analysis of total and phosphorylated forms of AKT and ERK1/2 in cell lysates prepared from T47D cells expressing shRNA specific for either PIP (shPIP/121, shPIP/214) or a non-genomic target (shNS).

The PIP-regulated, highly connected gene network (Figure 2D) included mRNAs that encode not only transcription factors such as cMYC and cJUN, but also secreted ligands such as TGF-beta-3 and CXCL12, as well as secondary messengers such as Janus kinase 1 (JAK1) and Rho family GTPase 3 (RND3). Plausibly, PIP affected expression of genes encoding ligands, kinases and their regulatory proteins, ultimately switching on major signaling cascades that control cell proliferation. Because receptor tyrosine kinases (RTKs) are excellent candidates for such major switches, we screened T47D cells for RTKs that are inhibited upon PIP silencing. To this end, we employed the RayBio® human RTK phosphorylation antibody array-1 to analyze RTK activation in T47D cells expressing either a non-specific shRNA and one that specifically knocked down PIP (shPIP/121; Figure 3A). Differential hybridization to the RayBio® array allows simultaneous semi-quantitative analysis of phosphorylated tyrosine residues in 71 different receptor tyrosine kinases (RTKs). The results demonstrated that PIP knockdown was associated with dramatic decreases in the phosphorylation of focal adhesion-kinase (FAK), ephrin-B3 (EphB3), FYN, and hemopoietic cell kinase (HCK) (Figure 3B, C). We independently confirmed the results from the antibody-array by western blot analysis of cell extracts prepared from dox- and vehicle-treated T47D/shPIP^dox^ cells using specific antibodies that detect FAK phosphorylation at the Tyr^397^ residue. As shown in Figure 3D, PIP silencing significantly reduced FAK phosphorylation at the Tyr^397^ position. As control, western analysis with a pan FAK antibody showed no significant effect of PIP silencing on the total FAK protein level (Figure 3D). As additional control, PIP knockdown affected neither phospho- nor total HGFR levels (Figure 3D). Furthermore, western analyses of the same extracts showed that PIP silencing resulted in decreased cMYC levels, without a compensatory increase in cMYC phosphorylation at the Thr^58^ and Ser^62^ residues (Figure 3D). Thus, PIP is required for the activation of multiple RTKs as well as the downstream expression and activation of cMYC.

Activation of RTKs could account for the reliance of T47D cells on PIP for proliferation [27]. Because these RTKs signal through phosphorylation of downstream kinases such as AKT and ERK1/2, we studied the influence of PIP on these kinases by western analysis with specific phospho-antibodies. Constitutive knockdown of PIP resulted in strong inhibition of AKT and ERK1/2 phosphorylation without affecting their total protein levels (Figure 3E). These results suggest that PIP is required for the activation of signaling events through RTKs (FAK, EphB3, HCK, FYN), and downstream serine-threonine kinases (AKT, ERK1/2).

### PIP plays a role in cell adhesion, cytoskeleton dynamics and protein secretion

**Figure 4:**
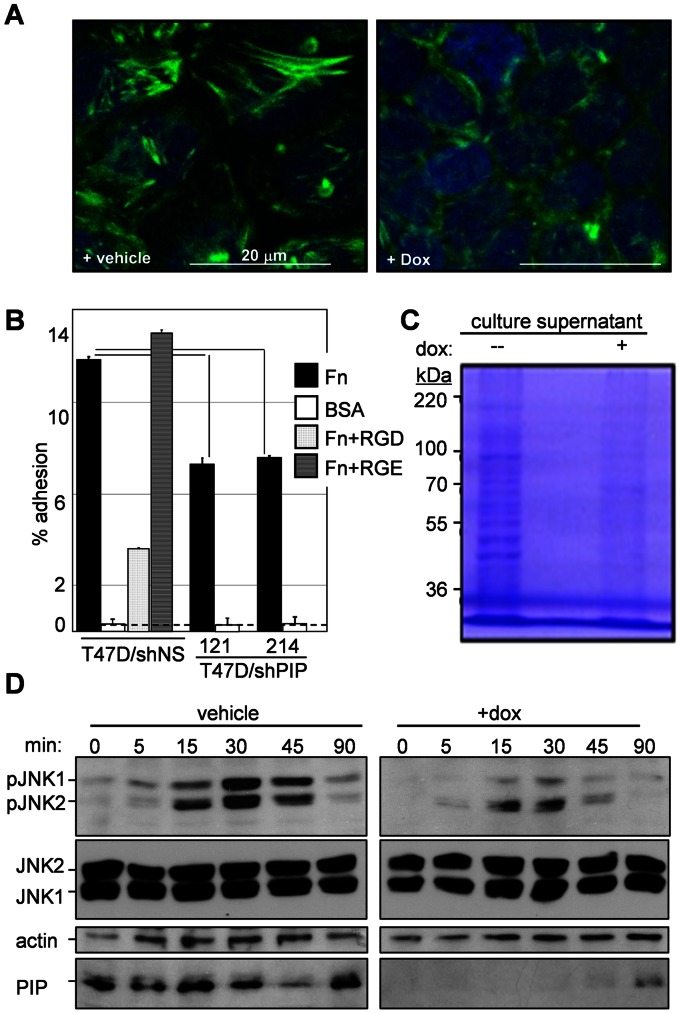
PIP is required for stress fiber assembly, cell adhesion, secretion, and JNK1 signaling in T47D BCa cells. **A,** Confocal images showing loss of phalloidin stained actin stress fibers in dox-treated *versus* vehicle-treated T47D/shPIP^dox^ cells. **B,** Adhesion assay showing inhibition of T47D cell adhesion to fibronectin after expression of PIP-specific (shPIP/121, shPIP/214) versus a non-specific (shNS) shRNA. Peptide competitors were used to assess specific (RGD) *versus* non-specific (RGE) binding to fibronectin. The dotted line indicates background binding to bovine serum albumin (BSA). **C,** Coomassie-stained SDS-polyacrylamide gel showing reduced levels of secreted proteins in the supernatant of dox- *versus* vehicle-treated T47D/shPIP^dox^ cell cultures. **D**, Western blot analysis of JNK1/2 phosphorylation in dox- *versus* vehicle-treated serum-starved T47D/shPIP^dox^ cells at the indicated time points after serum stimulation. Pan anti-JNK1/2 and tubulin antibodies were used as controls, and anti PIP antibodies were used to demonstrate effective dox-mediated PIP knockdown.

Defects in the phosphorylation of FAK, FYN and the downstream AKT and ERK1/2 are likely related to the inhibition of cell cycle progression observed after PIP knockdown in T47D cells [27]. We next investigated whether these defects were also associated with changes in cytoskeleton dynamics. T47D/shPIP^dox^ cells were treated with dox for two days to silence PIP, and stress fiber assembly was then induced by one-hour serum starvation and visualized by phalloidin staining and confocal microscopy. As shown in Figure 4A, a network of stress fibers was assembled in the control cells, but much less in the dox-treated, PIP-depleted cells. Because the actin cytoskeleton plays pivotal roles in cancer-related cellular processes such as adhesion and vesicular transport, we tested the effects of PIP silencing on these processes. *In vitro* adhesion assays indicated that PIP knockdown inhibited adhesion of T47D cells to a fibronectin matrix by 36–39% as compared to control cells (Figure 4B). We then compared the culture supernatants from control *versus* PIP-depleted T47D cell cultures. T47D/shPIP^dox^ cells were grown in culture medium containing vehicle or dox for two days to achieve effective PIP knockdown, and the cells were then washed and further incubated for 24 hours in serum-free medium. SDS-PAGE and coomasie blue staining of proteins precipitated from the conditioned media clearly indicated absence of many proteins from the medium conditioned by the dox-treated as compared to the vehicle-treated T47D/shPIP^dox^ cells (Figure 4C). Thus, the loss of stress fiber formation in PIP-depleted T47D BCa cells appears to inhibit both cellular attachment and protein secretion.

Because FAK-mediated stress fiber formation is controlled at least in part by JNK1 [28], and because JNK1 regulates expression of cMYC [23], [29], a major node in the PIP-activated genes network (Figure 2D), we studied the effect of PIP silencing on JNK1. T47D/shPIP^dox^ cells were maintained in serum-free medium for 24 hours in the presence or absence of dox, and JNK1 activation was assessed in cell extracts prepared 0, 5, 15, 30, 45, and 90 minutes after serum repletion. Western analysis with anti phospho-JNK1 antibodies showed that PIP knock-down diminished the phosphorylation of JNK1, but not the levels of total JNK1 or actin serving as controls (Figure 4D). Altogether, our data suggests that intracellular PIP regulates cytoskeleton dynamics, adhesion, secretion and proliferation of cancer cells via the phosphorylation of specific RTKs and downstream signaling pathways involving AKT, ERK1/2, JNK1 and cMYC.

### PIP is required for the proliferation of estrogen-dependent and tamoxifen-resistant T47D cells

**Figure 5:**
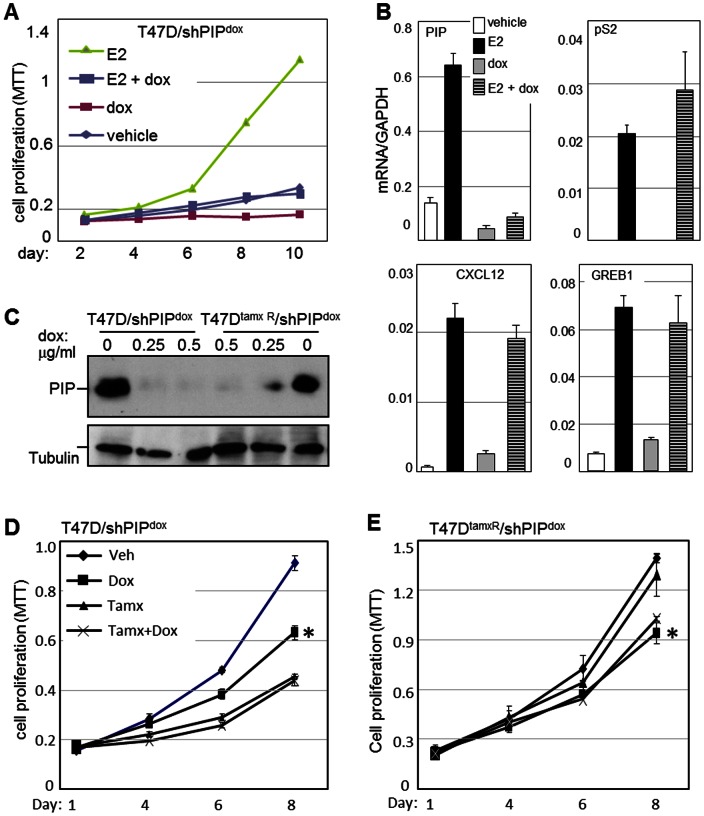
PIP is required for the proliferation of hormone-sensitive and –resistant breast cancer cells. **A,** The effect of PIP knockdown on E2-stimulated BCa cell proliferation was assessed in T47D/shPIP^dox^ cell cultures maintained in CSS-supplemented medium. Cells were treated with either E2 or vehicle in the presence or absence of dox, and MTT assays were performed every 48 hours for 10 days. **B,** RT-qPCR analysis of PIP and the estrogen-responsive genes pS2, CXCL12 and GREB1 in T47D/shPIP^dox^ cell cultures treated with E2 in the presence or absence of dox to silence PIP expression. Values are normalized per GAPDH expression, which itself did not significantly change by treatment with dox or E2. **C,** Immunoblot analysis showing the dox-induced PIP knockdown in the parental T47D and the tamoxifen-resistant T47D^tamxR^ cells, each engineered with a dox-inducible lentiviral vector encoding a small hairpin RNA that targets PIP (shPIP^dox^). **D–E,** MTT-based proliferation assays conducted after 4, 6, and 8 days of treatment with tamoxifen and/or dox, showing that PIP silencing inhibited proliferation of both the tamoxifen-sensitive T47D and the tamoxifen-resistant T47D^tamxR^ cells. (Mean±SD; n = 3). *significantly different from vehicle-treated cells at the day-6 and day-8 experimental time points (*p*<0.0004).

As estrogens are major culprits in BCa initiation and progression, and because luminal A-type tumors, which frequently express PIP, are usually ER-positive (Figure 1A), we investigated the requirement for PIP in estrogen-driven BCa cell proliferation. Equal numbers of T47D/shPIP^dox^ cells were plated and grown in medium containing 10% CSS as well as dox and/or E2. MTT-based assays were performed every 48 hours to assess cell proliferation. As shown in Figure 5A, cells proliferation was slower in CSS relative to complete serum (compare to Figure 1C–D). As expected, E2 treatment resulted in a strong mitogenic effect, and importantly this effect was almost completely antagonized by PIP knockdown. PIP also inhibited the low proliferative activity observed in the absence of steroid hormones (Figure 5A). To test whether PIP was required for estrogen signaling in T47D cells, we investigated the effect of PIP knockdown on E2-stimulated transcription of the estrogen receptor (ER)- alpha target genes pS2, GREB1 and CXCL12 [30], [31], [32]. T47D/shPIP^dox^ cells were grown in culture medium supplemented with CSS and either dox or vehicle for 48 hours. E2 or vehicle was then added for 24 hours, and the transcript levels of the ER-alpha target genes were analyzed by RT-qPCR. As shown in Figure 5B PIP knockdown did not compromise E2-mediated stimulation of any of the ER target genes tested. Thus, T47D BCa cells require PIP for proliferation in order to facilitate activity of a mitogenic pathway(s) that does not necessarily depend on estrogen-driven transcription.

Because PIP is required for the proliferation of both ER-positive and ER-negative BCa cells (Figure 1C, Figure S3 in File S1; [3]), and its knockdown did not influence estrogen-driven transcription (Figure 5B), we hypothesized that PIP silencing would be required for the proliferation of hormonal therapy-resistant BCa cells. To test this hypothesis, we employed T47D^tamR^ cells, a T47D-derived cell line rendered resistant to tamoxifen by continuous growth in estrogen-deprived culture medium [12]. A T47D^tamR^/shPIP^dox^ subline was engineered, which, like the T47D/shPIP^dox^ line, responds to dox treatment with expression of an shRNA that silences PIP expression. Western blot analysis indicated that T47D^tamR^ cells express PIP at comparable levels to those present in the parental T47D cells and that dox treatment reduced PIP expression in both of the engineered cell lines to barely detectable levels (Figure 5C). MTT-based assays were then conducted over time to compare the effects of PIP knockdown on T47D^tamR^/shPIP^dox^ and T47D/shPIP^dox^ cell proliferation. As control, tamoxifen treatment alone strongly inhibited the proliferation of T47D/shPIP^dox^ but not T47D^tamR^/shPIP^dox^ cells (Figure 5D, E). On the other hand, dox-induced PIP knockdown significantly inhibited the proliferation of both the T47D/shPIP^dox^ and the T47D^tamR^/shPIP^dox^ cells and the extents of inhibition were comparable at each of the experimental time points. Because PIP is required for the proliferation of both hormonal therapy-sensitive and -resistant BCa cells, its pharmacological inhibition may prove beneficial for patients at multiple disease stages.

## Discussion

Breast cancer (BCa) is a heterogeneous disease with multiple subtypes of tumors, some ER-alpha-positive (mostly luminal A, luminal B and normal-like), and others ER-alpha-negative (mostly HER2-enriched and basal-like). The estrogen and HER2 receptor-mediated pathways that primarily define these subtypes have been successfully targeted for treatment. However, frequent disease recurrence advocates the pursuit of new targets and therapeutic modalities to be used either alone or together with those currently available in order to achieve long-term survival and cure. The present study suggests that PIP, a small polypeptide expressed by the majority BCa tumors, may serve as a target for the development of novel therapeutic approaches for BCa. Knockdown of PIP in ER-positive T47D cells dramatically reduced proliferation independent of estrogen-driven transcription. Of note, over-expression of PIP in either PIP-positive T47D or PIP-negative MDA-MB-231 cultures had no effect on cell proliferation (Figure S3B in File S1 and data not shown). Unexpectedly, PIP supported cell proliferation via an intracellular mechanism because neither conditioned medium containing PIP nor co-culture with PIP-secreting cells rescued the proliferation defect caused by PIP silencing. These observations indicate a novel obligatory role for intracellular PIP in BCa cell proliferation. A screen of 71 receptor tyrosine kinases revealed that PIP silencing selectively reduced the tyrosine phosphorylation of FAK, FYN, EphB3, and HCK. Subsequent studies demonstrated defects in the activation of the downstream serine/threonine kinases AKT, ERK1/2, and JNK1. Furthermore, mRNA profiling followed by *in silico* analysis demonstrated an intricate PIP-controlled gene network with cMYC and cJUN, targets of JNK1 and ERK1/2, respectively, forming predominant regulatory nodes. Suggesting clinical significance of our *in vitro* data, expression of genes that were down-regulated upon PIP knockdown in T47D cell culture was significantly correlated with the levels of PIP mRNA across BCa tumors.

Even though PIP is most commonly expressed in ER-positive BCa tumors of the Luminal A-subtype, its requirement for cell proliferation is not unique to such cells. In fact, we have observed inhibition of cell proliferation upon PIP silencing in the ER-negative MDA-MB-453 cell line (Figure S3 in File S1), and this finding is consistent with the recent results of Naderi and Meyer in both MDA-MB-453 and HCC-1954 BCa cells [3]. Various mechanisms have been proposed to explain the requirement for PIP in the proliferation of various BCa cell subtypes. PIP was required for androgen signaling in the T47D BCa cell line [8], and its role in promoting MDA-MB-453 cell proliferation was related to activation of the HER2 receptor tyrosine kinase. Notably, HER2 is not over-expressed in the ER-positive T47D cells ([33]; Figure 3B) and our RTK screen suggested that other RTKs (FAK, FYN, EphB3, HCK) mediated the functions of PIP in these cells. We speculate that PIP plays a common fundamental function in various types of cancer cells, which utilize PIP to promote different pathways with crucial roles in the different cell types. Furthermore, promotion of BCa cell proliferation by various hormones and growth factors may require the stimulation of PIP. Indeed, such stimulation has been observed in response to estrogens (Figure 5B; [34], [35]), androgens, prolactin and growth hormone [3], [4], [5], [9]. The identity and flexible nature of a common PIP function operative as an obligatory component of various growth stimulatory networks remains to be explored. Thus, inhibition of PIP may serve as a therapeutic strategy to abrogate the adhesion and growth of BCa cells across multiple tumor subtypes. Furthermore, although PIP expression typifies ER-alpha positive BCa, future therapeutic approaches that target PIP-related mechanisms may be effective for the treatment of both ER-positive and –negative BCa cell lines (Figure S3A in File S1). The insensitivity of some BCa cell lines to PIP, however (Figure S3B in File S1), suggest that such future therapeutic approaches will not be universally efficacious.

Inhibition of PIP may prove effective for the treatment of BCa cells resistant to hormonal therapy, because it's silencing equally inhibited proliferation of the tamoxifen-resistant T47D^tamR^ cells and the naïve tamoxifen-sensitive T47D cells. The clinical significance of this observation is emphasized by reports that PIP expression is strongly enhanced upon tamoxifen treatment [34], [35], and that drug-resistant BCa cells continue to express PIP (Figure 5C). Targeting PIP may therefore prove effective not only in early disease stages, but also in advanced tumors resistant to hormonal therapy.

In summary, our studies assign essential roles to PIP in both hormone-responsive and -unresponsive breast cancer cells, thereby extending the importance of PIP in the management of BCa from a mere clinical marker to a promising therapeutic target. Finally, the design of PIP inhibitors for the treatment of breast and other cancers will have to take into consideration our findings, suggesting a novel intracellular role that PIP plays in cancer cell proliferation independently of the roles that it plays as a secreted protein.

## Supporting Information

File S1 **Supplemental figures.**
(PDF)

Table S1 **Oligonucleotides used in this study.**
(DOC)

Table S2 **Dox regulated at D1 or D2.**
(XLS)

Table S3 **Day 1 1.5 fold repressed 293 genes.**
(XLS)
