# Supplementary material for: Prolactin-Induced Protein (PIP) Regulates Proliferation of Luminal A Type Breast Cancer Cells in an Estrogen-Independent Manner
Source: PLoS One. 2013 Jun 3;8(6):e62361. doi: 10.1371/journal.pone.0062361 (PMC3670933; doi:10.1371/journal.pone.0062361)
Supplement: File S1 — Supplemental figures. (PDF) [file pone.0062361.s001.pdf]

Figure S1

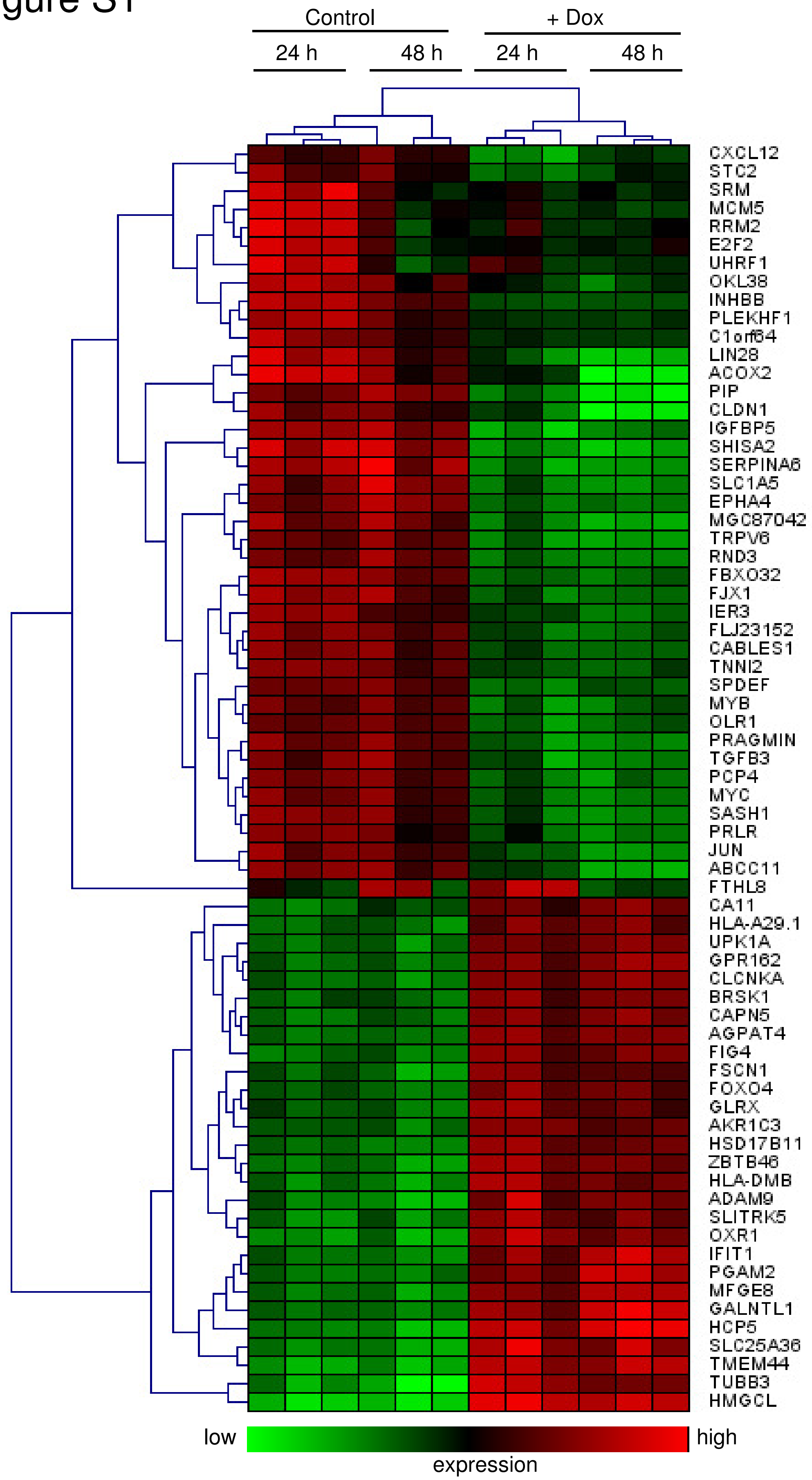

Figure S1 **Unsupervised hierarchical clustering of differentially expressed genes**. Intensity profile of genes that showed ≥2-fold up- or down-regulated expression on either day 1 or day 2 (total: 69 genes) with $p < 0.04$. Genes were subjected to pearson's centered correlation matrix using the Genesis software tool from the Institute for Genomics and Bioinformatics at Graz University of Technology, Switzerland. Heatmap represents intensity values relative to the median intensity across all 12 samples per probe after background subtraction and normalization.

Figure S2

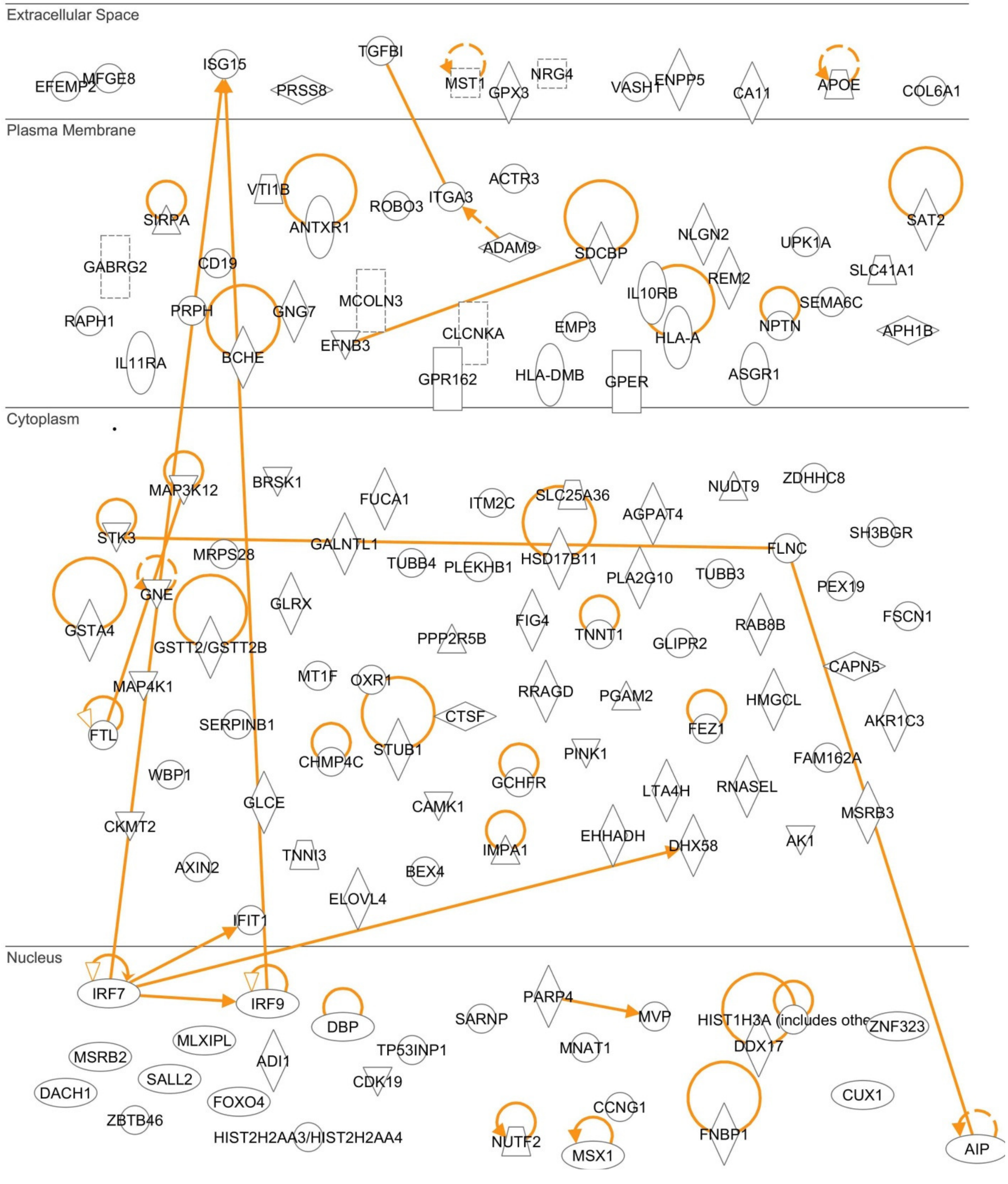

Figure S2 **Pathway analysis of genes up-regulated by PIP knockdown.** 273 genes up-regulated by ≥1.5-fold one day after PIP knockdown were subjected to the pathway analysis tool from Ingenuity Systems (IPA™). Connections are shown among 132 genes for which matched entries were found. Each connection indicates at least one direct relationship found in the literature.

.

Figure S3

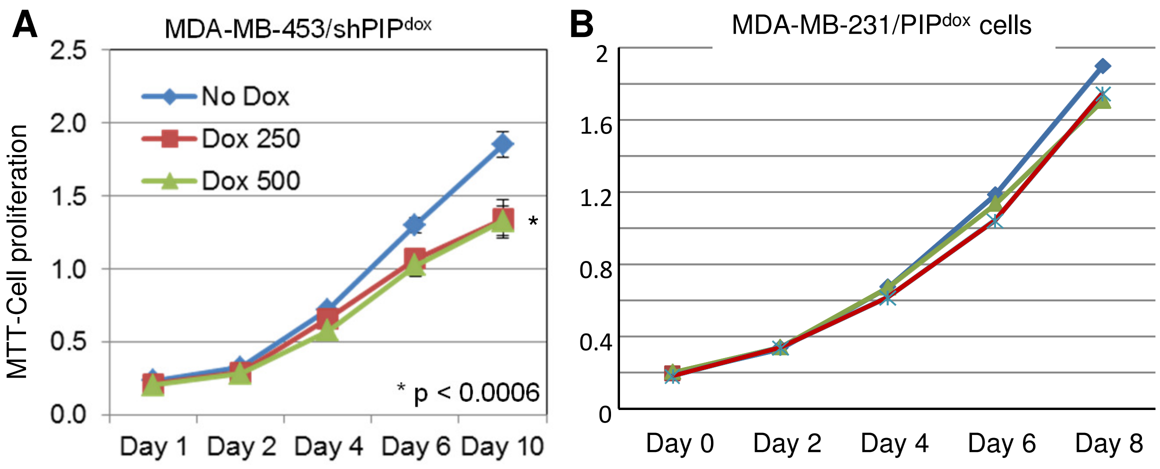

Figure S3 **PIP is required for the proliferation of ER-negative MDA-MB-453 cells**. Expression of PIP was either knocked-down in PIP-positive MDA-MB-453 cells **(A)** or induced in PIP-negative MDA-MB-231 cells **(B)** using lentiviral particles encoding shPIP^{dox} or PIP^{dox}, respectively. Cell proliferation was assessed using MTT assays at the indicated time points. Cells cultures were treated with either 250 ng/ml or 500 ng/ml of Dox, and an equal volume of distilled water was used as vehicle control.

Figure S4

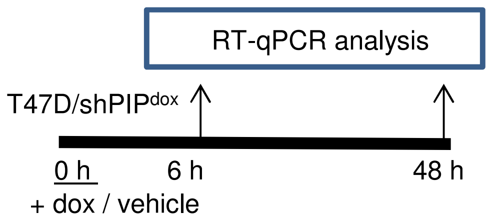

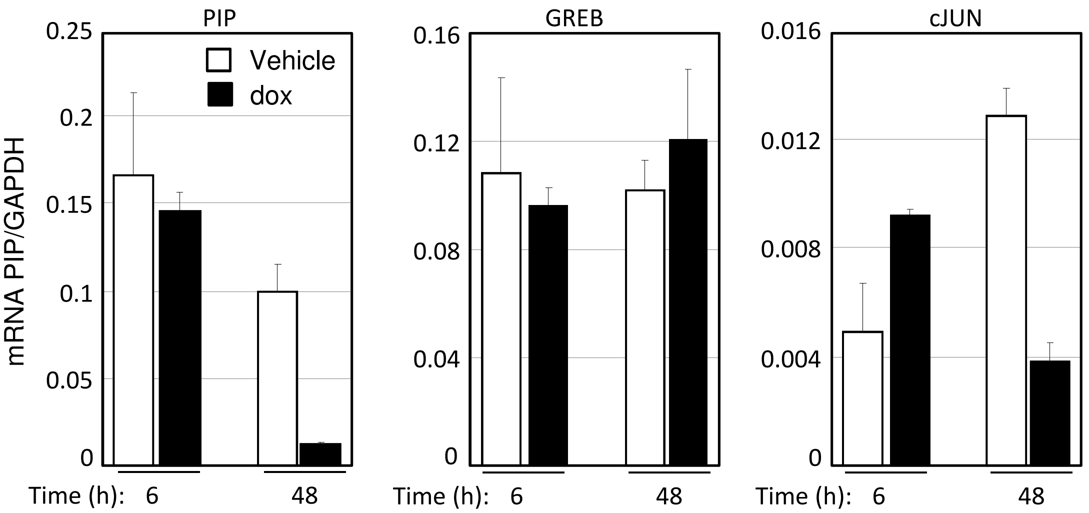

Figure S4 **RT-qPCR analysis of PIP and its target cJUN after PIP knockdown.** RNA was extracted after 6h and 48h treatment of T47D/shPIP^{dox} cells with either dox (250 ng/ml) or vehicle as schematically illustrated in the upper panel. Expression levels of PIP, GREB, and cJUN were measured relative to GAPDH as control (lower panel). The result demonstrate that Dox-induced shPIP expression decreased PIP and increased cJUN mRNA levels at the 48, but not at the 6-hour time point. RTqPCR analysis of GREB is presented as negative control.
