# Supplementary material for: Prolactin-Induced Protein (PIP) Regulates Proliferation of Luminal A Type Breast Cancer Cells in an Estrogen-Independent Manner
Source: PLoS One. 2013 Jun 3;8(6):e62361. doi: 10.1371/journal.pone.0062361 (PMC3670933; doi:10.1371/journal.pone.0062361)
Supplement: Table S1 — Oligonucleotides used in this study. (DOC) [file pone.0062361.s002.doc]

| **Table S1: Oligonucleotides used in this study** | | |
| --- | --- | --- |
| RT-qPCR primers | Forward | Reverse |
| PIP | GTACGTCCAAATGACGAAGTCAC | CAGCAGCATCATCAGGGCAGATG |
| GAPDH | gtcatgggtgtgaaccatgaga | ggtcatgagtccttccacgatac |
| pS2 | GAACAAGGTGATCTGCG | TGGTATTAGGATAGAAGCACCA |
| CXCL12 | TCAGCCTGAGCTACAGATGC | CTTTAGCTTCGGGTCAATGC |
| GREB1 | CAAAGAATAACCTGTTGGCCCTGC | GACATGCCTGCGCTCTCATACTTA |
| **shPIP^dox^ Cloning Oligonucleotides** | | |
| 21 mer Target sequence in ORF of PIP: CCTGCCTATGTGACGACAATC | | |
| Fwd Oligo | AGCGA**CCTGCCTATGTGACGACAATC**TAGTGAAGCCACAGATGTAGATTGTCGTCACATAGGCAGGC | |
| Rev. Oligo | ggcaG**CCTGCCTATGTGACGACAATC**TACATCTGTGGCTTCACTAGATTGTCGTCACATAGGCAGGT | |
| 21 mer Target sequence in the 3’UTR of PIP: GATTTCCTCTAAAGAAACTTG | | |
| Fwd Oligo | AGCGCGATTTCCTCTAAAGAAACTTGTAGTGAAGCCACAGATGTACAAGTTTCTTTAGAGGAAATCA | |
| Rev. Oligo | GGCATGATTTCCTCTAAAGAAACTTGTACATCTGTGGCTTCACTACAAGTTTCTTTAGAGGAAATCG | |
| Constitutive ShPIP/121 obtained from SIGMA (TRCN0000137374): GACATTCCCAAGTCAGTACG | | |
| Constitutive ShPIP/214 obtained from SIGMA (TRCN0000136808) CTCATTAGCAGCATCCC | | |
|  | | |
